# Fucoxanthin induces apoptosis and reverses epithelial-mesenchymal transition via inhibiting Wnt/β-catenin pathway in lung adenocarcinoma

**DOI:** 10.1007/s12672-022-00564-4

**Published:** 2022-10-03

**Authors:** Heqi Luan, Lina Yan, Yuanyuan Zhao, Xuejiao Ding, Lihua Cao

**Affiliations:** Department of Respiratory Medicine, Τhe Second Hospital Affiliated to Dalian Medical University, No. 467 Zhongshan Road, Shahekou District, Dalian, 116027 Liaoning People’s Republic of China

**Keywords:** Fucoxanthin, Non-small cell lung cancer, Apoptosis, Epithelial-mesenchymal transition

## Abstract

**Background:**

Invasion and metastasis are hallmark characteristics of cancer and the main causes of death in cancer patients. Studies have shown that epithelial-mesenchymal transition (EMT) plays significant role in tumor invasion and metastasis. Fucoxanthin, a carotenoid found in seaweeds, has been proved to have anti-tumor effects. Our study aimed to research the role of fucoxanthin on proliferation, apoptosis, migration and EMT of two types of LUAD cells.

**Methods:**

Cell migration and invasion were examined by Wound-healing and Transwell assays. Western blot assay was used to detect the expression levels of apoptosis-related proteins, EMT-related proteins and β-catenin. Immunohistochemistry was used to detect the expression of β-catenin in human lung adenocarcinoma tissues and corresponding para-cancerous tissues.

**Results:**

Our results revealed that fucoxanthin depressed the proliferation and induced apoptosis in A549 and NCI-H1299 cells. Moreover, fucoxanthin reversed TGF-β1-induced EMT and cell motility. Meanwhile, we disclosed that fucoxanthin and XAV939 had similar effect on β-catenin, EMT protein and cell motility. What is more, immunohistochemical results revealed that the high expression rate and abnormal expression rate of β-catenin in cancer tissues was significantly higher than that in para-cancerous tissues.

**Conclusion:**

Taken together, the findings of our research highlight a novel role for fucoxanthin in NSCLC cells, which might be a potentially effective anti-tumor agent for the treatment of LUAD patients.

**Supplementary Information:**

The online version contains supplementary material available at 10.1007/s12672-022-00564-4.

## Background

Lung cancer is the main cause of cancer morbidity and mortality worldwide, and lung cancer patients accounted for approximately 18.4% of cancer deaths in 2018 [1]. Lung cancer is divided into small cell lung cancer (SCLC) and non-small cell lung cancer (NSCLC). About 85% of lung cancer is NSCLC, and lung adenocarcinoma (LUAD) is the main pathological type of NSCLC [2]. Radical resection is considered to be the preferred treatment for patients with early-stage lung adenocarcinoma, but there is still a certain risk of recurrence [3] and distant metastasis [4]. Therefore, identifying patients with high-risk recurrence and metastasis and investigating new effective ingredients for NSCLC patients will be of great significance to the prognosis of patients.

Invasion and metastasis of cancer cells is the main cause of cancer death [5]. Epithelial-mesenchymal transition (EMT) is considered a prerequisite of tumor invasion and metastasis [6]. EMT is the result of the interaction of transcription factors (such as Snail, Zeb and Twist) and post-transcriptional regulators. The main characteristics of EMT are the loss of cell adhesion, including the down-regulation of epithelial markers (E-cadherin, etc.) and the up-regulation of mesenchymal markers (vimentin, N-cadherin, etc.) [7]. At the same time, recent studies have shown that EMT is associated with lung tumor susceptibility and is an early event in the pathogenesis of lung tumor [8]. Therefore, understanding the underlying molecular mechanisms regulating lung cancer metastasis and the corresponding targeted therapy are helpful to the diagnosis and treatment of lung cancer.

The Wnt/β-catenin signaling pathway is essential for embryonic development and cancer development. Without Wnt stimulation, β-catenin is degraded by proteins that includes adenomatous polyposis coli (APC), glycogen synthase kinase 3β (GSK3β) and Axin. The binding of Wnt ligands with Frizzled and Low-density lipoprotein receptors (LRP5 and LRP6) inhibits the activity of the complex, resulting in the accumulation of nuclear β-catenin to interact directly with T-cell transcription factor (TCF) and other factors to regulate transcription [9]. At present, a large number of researches have shown that altered activity of the Wnt/β-catenin signaling pathway participates in the process of EMT and cancer metastasis [10]. Therefore, understanding how the Wnt/beta-catenin signaling is regulated will have a certain reference value in the research of anti-metastasis drugs.

Fucoxanthin is a carotenoid, which mainly exists in algae [11]. Some studies have reported that fucoxanthin exerts important biological effects, such as anti-tumor [12], anti-inflammatory [13], anti-oxidation [14] and antidiabetic [15] activity. Fucoxanthin also has been shown that it can promote apoptosis in lung cancer [16], colorectal cancer [17], breast cancer [18], liver cancer [19], gastric cancer [20], but its role in invasion and metastasis of cancer cells has rarely been investigated. The present experiment was conducted to investigate the efficacy of fucoxanthin on LUAD cells and its underlying molecular mechanism, hoping to provide a reference for the treatment of lung cancer patients.

## Material and methods

### Cell culture

Human LUAD cell lines A549 and NCI-H1299 were obtained from the Cell Bank of Chinese Academy of Sciences (Shanghai, China). Cells were cultured in RPMI-1640 medium (Gibco, Invitrogen; Thermo Fisher Scientific, Inc., Carlsbad, CA, USA) containing 10% fetal bovine serum (FBS; Thermo Fisher Scientific, Inc.) and 1% penicillin/streptomycin (Gibco, Grand Island, NY, USA) at 37˚C in a humidified atmosphere containing 5% CO_2_.

### Reagents

Fucoxanthin purchased from MedChemExpress (New Jersey, USA) (purity 99.17%) was dissolved in dimethyl sulfoxide (DMSO) to make a 50 mM stock and stored at -20℃ and diluted with fresh culture medium before use. An equal volume of DMSO (final concentration < 0.1%) as added to the controls. TGF-β1 was purchased from PeproTech (Rocky Hill, NJ, USA). Primary monoclonal antibodies including rabbit anti-human Bax, Bcl-2, Caspase 3, Cleaved Caspase 3, PARP, Cleaved PARP, E-cadherin, Vimentin, β-catenin, and glyceraldehyde-3-phosphate dehydrogenase (GAPDH) antibodies were purchased from ProteinTech Group, Inc. (Chicago, IL, USA). The Alexa Fluor® 790-conjugated donkey anti-rabbit secondary antibodies were purchased from Abcam (Cambridge, MA).

### Cell viability assay

The effects of fucoxanthin on cell viability was detected using Cell Counting Kit-8 (CCK-8, Dojindo Laboratories, Kumamoto, Japan) on the basis of the manufacturer's instructions. Cells were inoculated in 96‐well plates at a density of 5000/well and preincubated for 24 h in 37℃, 5% CO_2_ incubator. Three groups (control group, experimental group and blank group) were designed with five copies each group. Afterwards, cells were treated with fucoxanthin at the concentration of 12.5, 25, 50, 75, 100 μM for 12, 24, 48, 72 h. Then, 10 μl CCK-8 solution was added to each well, and cultured at 37℃ for 2 h. After that, the absorbance at 450 nm was detected using a microplate reader (Thermo Fisher Scientific, Carlsbad, CA, USA). A microplate reader (Bio-Rad Laboratories, Hercules, CA, USA) was used to measure the amount of formazan dye (450 nm absorbance), which is directly proportional to the number of living cells. Cell proliferation inhibition rate was calculated is as follows: (OD control – OD experiment)/(OD control − OD blank).

### Wound-healing assay

Human LUAD cell lines A549 and NCI-H1299 cells (5 × 10^5^ /well) were seeded in 6-well plates and cultured for 24 h. After 6 h of starvation in serum-free medium, a scratch was made in each well using a sterile 200 µl pipette tip, and cellular debris was removed with PBS. Thence, the cells were cultured in serum-free medium containing 10 ng/ml TGF-β1 and/or different concentrations of fucoxanthin, 10 μM XAV939 for 48 h. Migration distance was measured by an inverted microscope at the indicated times (0 and 48 h).

### Transwell assay

Cell culture inserts (8 µm; Costar, Corning, NY, USA) were used in a Transwell assay to investigate the invasive behavior of A549 and NCI-H1299 cells. The upper chambers were coated with 60 µl Matrigel gel (Becton, Dickinson and Company, Franklin, Lakes, NJ, USA) for 2 h. NSCLC cells in 200 μl of serum-free medium containing TGF-β1 (10 ng/ml) and/or fucoxanthin (25 μM or 50 μM), XAV939 (25 μM) were placed in the upper chamber, and complete medium containing 10% FBS was added to the lower chamber. After 48 h, cells on the upper surface of the insert were gently removed with a cotton swab. Cells that had migrated were fixed with 4% paraformaldehyde and then stained with 0.1% crystal violet for 10 min. Microscope was used to count the average number of migrating cells.

### Western blot analysis

Cells were treated with TGF-β1 (10 ng/ml) and/or fucoxanthin (25 μM or 50 μM), XAV939 (25 μM) for 48 h, then lysed in radioimmunoprecipitation assay buffer (Cell Signaling Technology, Danvers, MA, USA) for 30 min. Next, the samples were centrifuged at 12000rmp for 20 min on ice to obtain the supernatant. The concentration of total protein was determined by a BCA protein assay kit (Abcam, Cambridge, UK). Equal amounts of proteins were separated by sodium dodecyl sulfate–polyacrylamide gel electrophoresis (SDS-PAGE) and transferred to polyvinylidene difluoride (PVDF) membranes. After blocking with 5% nonfat milk in TBST for 1 h at room temperature, the membranes were incubated with primary rabbit monoclonal antibodies (dilution 1:500) at 4 ℃ overnight. Membranes were then repeated washed and incubated with Alexa Fluor® 790-linked secondary antibodies (dilution 1:10000). Finally, images were captured with Odyssey Infrared Imaging System, and the densitometry of each protein band was quantified using ImageJ software.

### Clinical data

We collected 38 lung tumor tissues and corresponding non-tumor tissues from patients with lung adenocarcinoma who underwent thoracic surgery in our hospital from December 2018 to March 2019. The present study was conducted according to the guidelines of the National Institutes of Health and was approved by the Ethical Committee of the Second Affiliated Hospital of Dalian Medical University (Approval ID: 0120181115), and all the patients fully understood this study and signed the informed consent. Moreover, none of the participants received any antineoplastic treatment before operation. The average age of the patients was 61.36 ± 8.93 years old. There were 16 male patients and 22 female patients. The clinical diagnosis was based on histopathology. The clinical stages were stages I-II. Among them, 4 cases were poorly differentiated and 34 cases were moderately or highly differentiated. The number of cases with tissue size < 3 cm was 30, and 8 cases with tissue size ≥ 3 cm.

### Immunohistochemistry

All tissues were fixed in 5% formalin for 24 h at room temperature, and then the paraffin‑embedded tissues were sectioned (4 µm) and placed in a thermostatic oven at 65 ˚C overnight. Subsequently, the slides were deparaffinized and rehydrated by using xylene and graded ethanol. Antigen retrieval was performed by placing the slides in citrate buffer at 100 ˚C for 20 min. Immunohistochemistry staining was performed by using Streptavidin-Peroxidase IHC assay kit (ZSGB-bio, China). Then, the slides were incubated with primary antibodies against β-catenin (dilution 1:50) overnight at 4 ˚C. The second antibody was added to the tissues and incubated at 37 ˚C for 20 min. The slices were washed with PBS buffer three times, and DAB color solution was dripped to observe the pornography under the microscope.

Immunostaining results were judged by two pathologists independently. The β-catenin staining intensity was observed under a microscope. Semi-quantitative method was used to analyze the staining of β-catenin. Firstly, the staining intensity was scored as 0, 1, 2 and 3 points of blank, weak, moderate, and strong. Then, the percentages of positive cells were scored: 0 points (0–9%), 1 point (10–24%), 2 points (25–50%), and 3 points (51–100%). Finally, the multiplication of these two scores was used as the final score and grading. These scores (≥ 5) was defined as high expression, while scores (< 5) was defined as low expression. The expression of β-catenin was defined as the normal expression when it was over 70% expression on the cell membrane, and less than 70% expression was defined as low expression or loss expression. When the expression of β-catenin in cytoplasm and/or nucleus is more than 10%, it is defined as ectopic expression. Low or loss expression and ectopic expression were considered as abnormal expression [21].

### Statistical analysis

The SPSS 25.0 software (IBM Corp., Armonk, NY, USA) was used to analyze the data. Data were expressed as mean ± standard deviation. Student's t‑test and analysis of variance (one-way ANOVA) were used to assess differences between groups. The chi-square test was used to assess IHC results. And p-value < 0.05 or p-value < 0.01 indicates that the differences between groups have statistical significance.

## Results

### Fucoxanthin suppresses LUAD cells proliferation

**Fig. 1:**
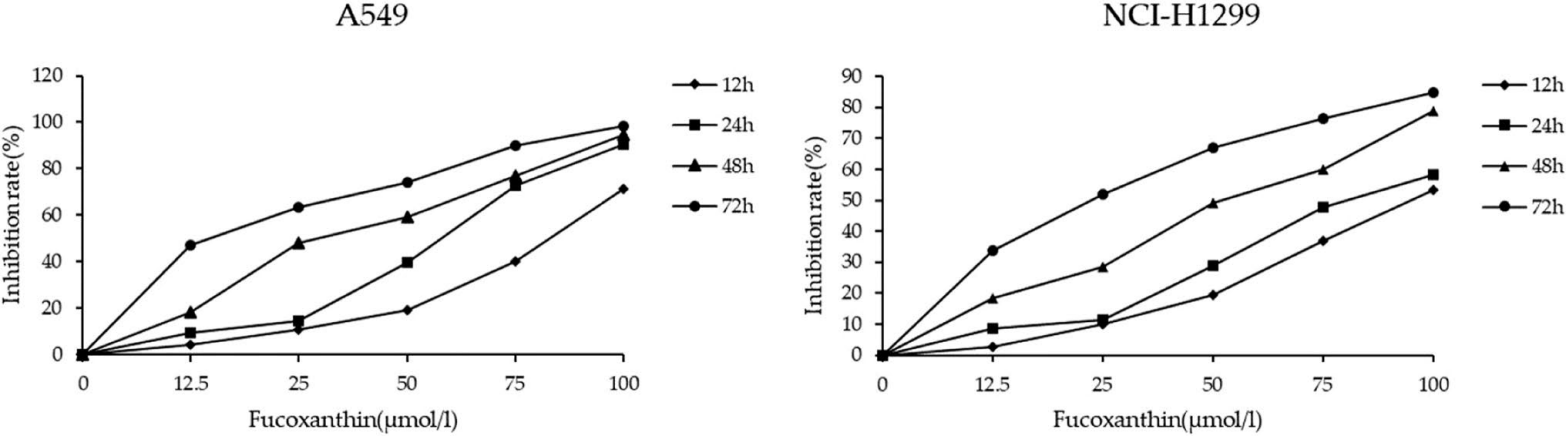
Effect of fucoxanthin on the A549 and NCI-H1299 cells viability. Cells were incubated with fucoxanthin (12.5, 25, 50, 75, 100 μM) for 12, 24, 48 and 72 h respectively. The inhibitive rates of A549 and NCI-H1299 cells were determined by Cell Counting Kit‐8 assay

The CCK-8 results showed that fucoxanthin could inhibit the proliferation of A549 and NCI-H1299 cells and present both dose and time dependent approximately (Fig. 1). The half inhibition concentrations (IC50) of fucoxanthin at 48 h were 25 μM for A549 cells, and 50 μM for NCI-H1299 cells, and these concentrations were used for the following study.

### Fucoxanthin promotes LUAD cells apoptosis

**Fig. 2:**
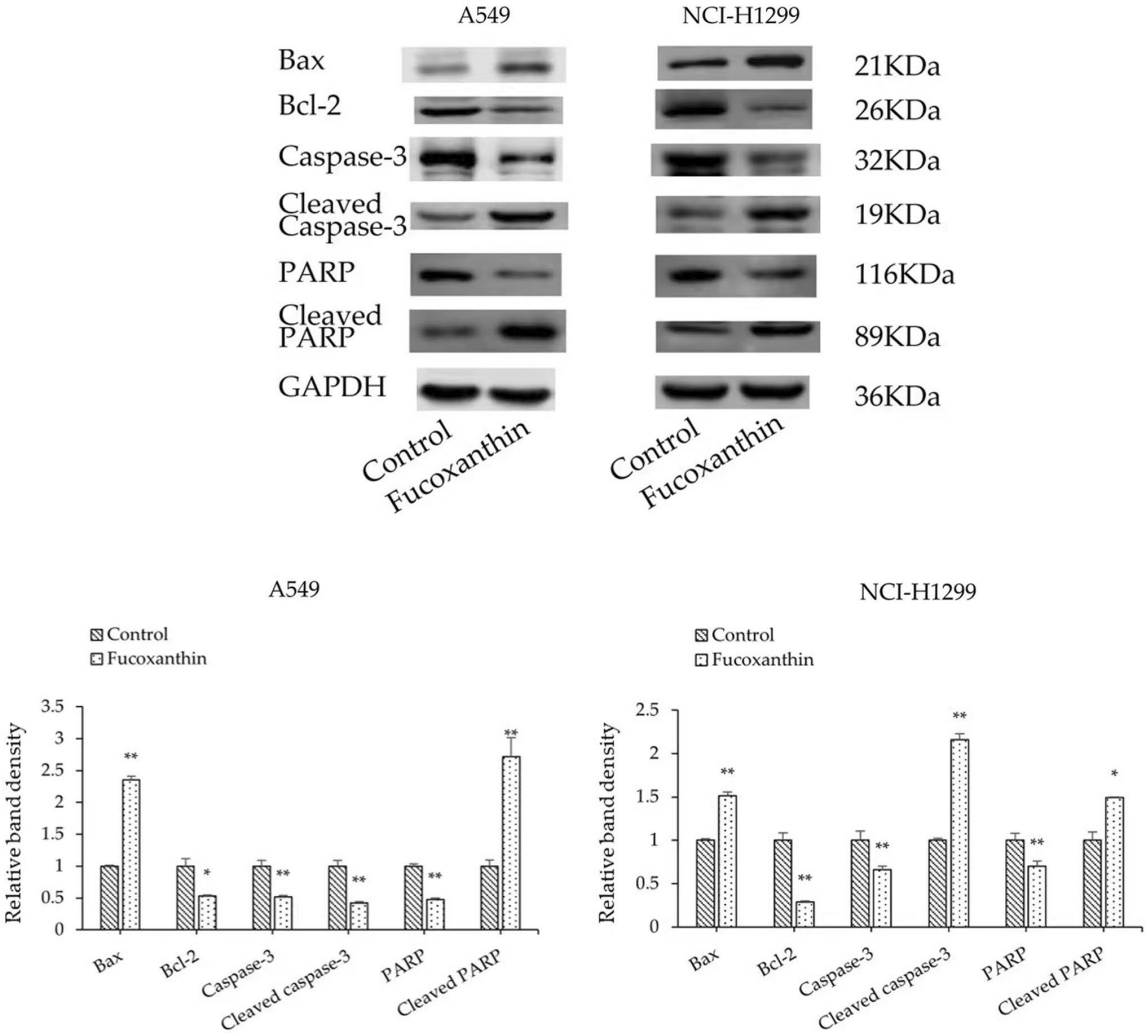
Effect of fucoxanthin on apoptosis-related proteins. A549 and NCI-H1299 cells were respectively exposed to 25 μM and 50 μM fucoxanthin for 48 h. Western Blot analysis was used to evaluate the apoptosis‐related proteins Bax, Bcl-2, Caspase 3, Cleaved caspase 3, PARP and Cleaved PARP of the cells treated as above. *P < 0.05, **P < 0.01 compared to control group. Data were expressed as mean ± standard deviation. One-way ANOVA were used to assess differences between groups. The SPSS 25.0 software (USA) was used to create the figures

Since the cell viability was efficiently restrained after treating with fucoxanthin, apoptosis-related proteins were detected to clarify the effect of fucoxanthin on cell apoptosis 48 h after fucoxanthin treatment. As shown, protein levels of Bax, Cleaved caspase 3, Cleaved PARP increased while those of Bcl-2, Caspase 3 and PARP decreased (Fig. 2).

### TGF-β1 regulates migration, invasion and EMT of LUAD cells

**Fig. 3:**
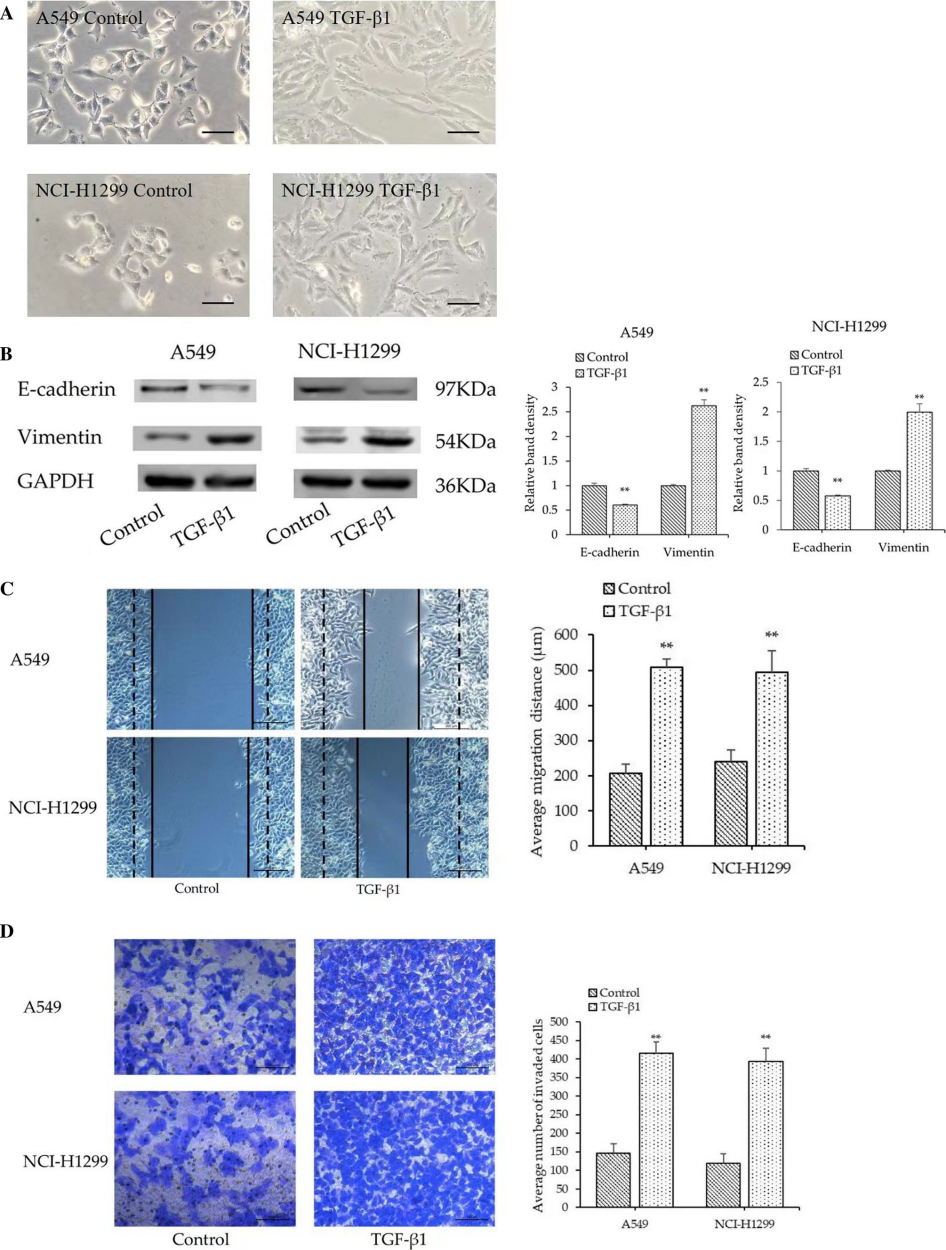
TGF-β1 regulates morphology migration, invasion and EMT of A549 and NCI-H1299 cells. **A** Micrographs of A549 and NCI-H1299 cell morphology after their culture in 10 ng/ml TGF-β1 for 48 h (Magnification, × 100). **B** The expression of E-cadherin and vimentin was evaluated by Western Blot analysis. **C** Wound healing assay (Magnification, × 100) and **D** Transwell assay (Magnification, × 200) were performed to assess the cell migration and invasion abilities. ** *P* < 0.01 compared to control group. Data were expressed as mean ± standard deviation. One-way ANOVA were used to assess differences between groups. The SPSS 25.0 software (USA) used for creation of the figure

Following exposure to 10 ng/ml TGF-β1 for 48 h, two types of cells exhibited typical cell morphological changes by marked stretching and elongation (Fig. 3A). Western blot analysis was first conducted to investigate the expression of the epithelial marker (E‐cadherin) and mesenchymal marker (Vimentin) in NSCLC cancer cells. The results showed that E‐cadherin level was significantly decreased, whereas expression of vimentin was increased in A549 and NCI-H1299 cells (p < 0.05; Fig. 3B). We next investigated the effects of TGF-β1 on the migration and invasion of NSCLC cells. As the wound healing assay and Transwell assay showed (Fig. 3C and D), the migration distances and the number of invaded cells were obviously increased in the TGF-β1 group.

### Fucoxanthin reverses cell migration and invasion and EMT induced by TGF-β1

**Fig. 4:**
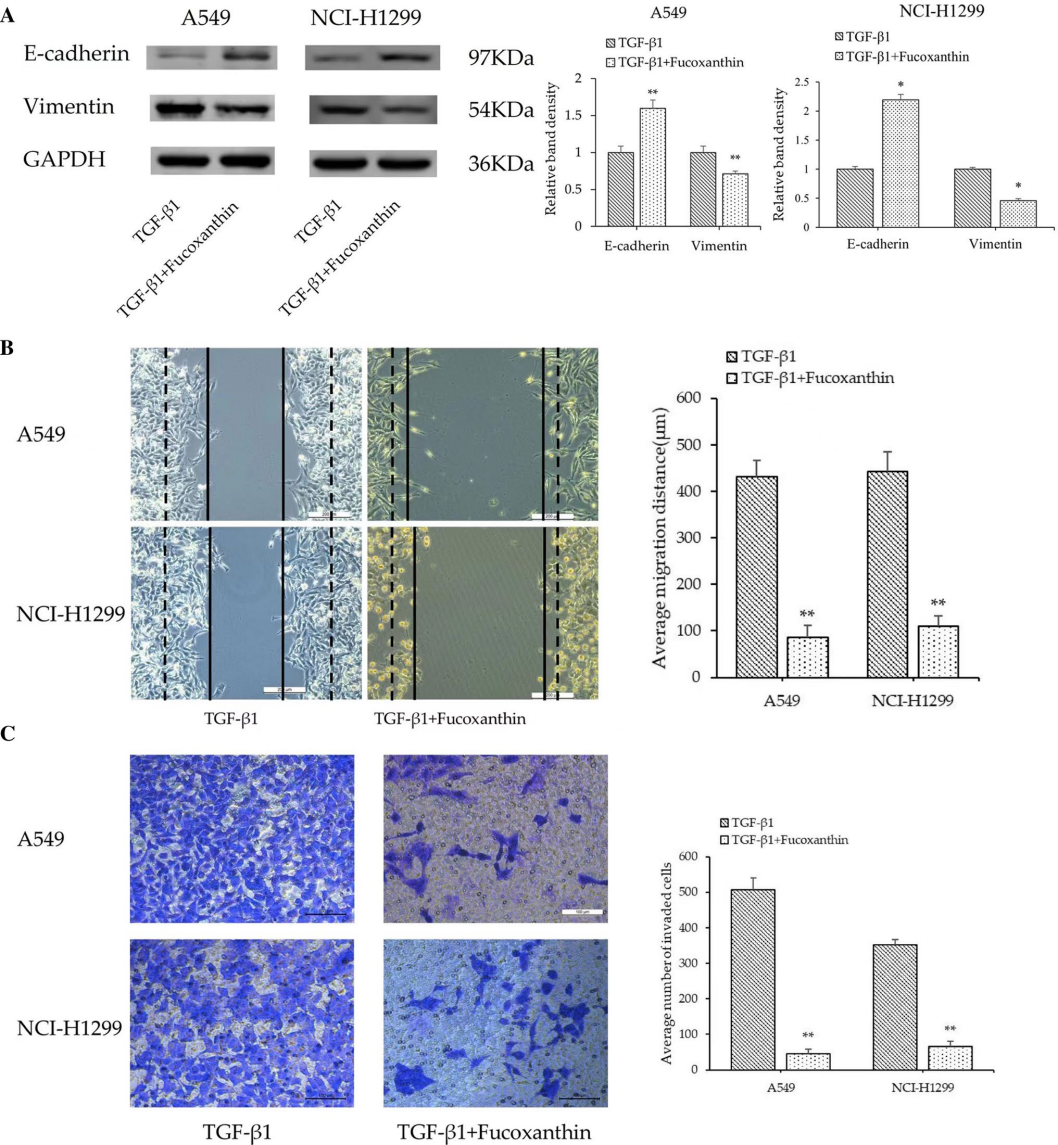
Fucoxanthin reverses cell migration and invasion and EMT induced by TGF-β1. Cell lines were incubated with TGF-β1 (10 ng/ml) and/or fucoxanthin (25 μM or 50 μM) for 48 h. **A** The levels of EMT-related protein were evaluated by Western Blot analysis. **B** Wound healing assay (Magnification, × 100) and **C** Transwell assay (Magnification, × 200) were performed to assess the cell migration and invasion abilities. **P < 0.01 compared to TGF-β1 group. Data were expressed as mean ± standard deviation. One-way ANOVA were used to assess differences between groups. The SPSS 25.0 software (USA) used for creation of the figure

We analyzed the influence of fucoxanthin on EMT of NSCLC cells pretreated with TGF-β1. As shown in Fig. 4A, compared with the TGF-β1 group, fucoxanthin‐treated cells had increased E‐cadherin expression, whereas the expression of vimentin was reduced. Moreover, compared with the TGF-β1 group, fucoxanthin led to significantly decreased migration distances and numbers of invasive cells (*P* < 0.01; Fig. 4B, C).

### Fucoxanthin blocks the formation of EMT by inhibiting Wnt/β-catenin signaling pathway

**Fig. 5:**
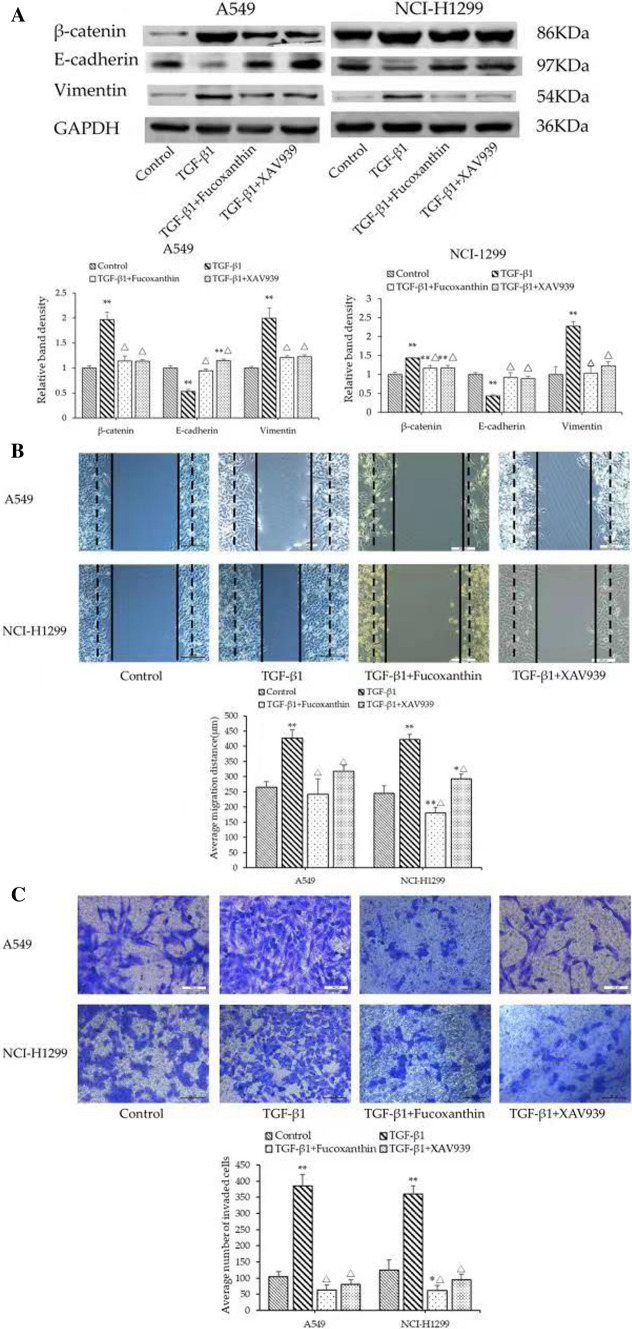
Fucoxanthin reverses EMT and cell motility by inhibiting Wnt/β-catenin signaling pathway. Cell lines were incubated with TGF-β1 (10 ng/ml) and/or fucoxanthin (25 μM or 50 μM), XAV939 (25 μM) for 48 h. **A** The levels of EMT-related protein were evaluated by Western Blot analysis, as indicated. **B** Wound healing assay (Magnification, × 100) and **C** Transwell assay (Magnification, × 200) were performed to assess the cell migration and invasion abilities. *P < 0.05, **P < 0.01 compared to control group, △*P* < 0.05 compared to TGF-β1 group. Data were expressed as mean ± standard deviation. One-way ANOVA were used to assess differences between groups. The SPSS 25.0 software (USA) used for creation of the figure

To further elucidate the underlying molecular mechanism that fucoxanthin acts on EMT in NSCLC cells, we measured the Wnt/β-catenin signaling pathway during TGF-β1 and fucoxanthin interference. Western blotting results confirmed that TGF-β1 promoted the expression of β-catenin while fucoxanthin led to significantly decreased expression in A549 and NCI-H1299 cells (Fig. 5A). Meanwhile, we disclosed that the effect of Wnt/β-catenin pathway inhibitor XAV939 on β-catenin, EMT protein and cell motility was consistent with that of fucoxanthin (Fig. 5A–C).

### The expression of β-catenin in lung cancer and para-cancerous tissues

**Fig. 6:**
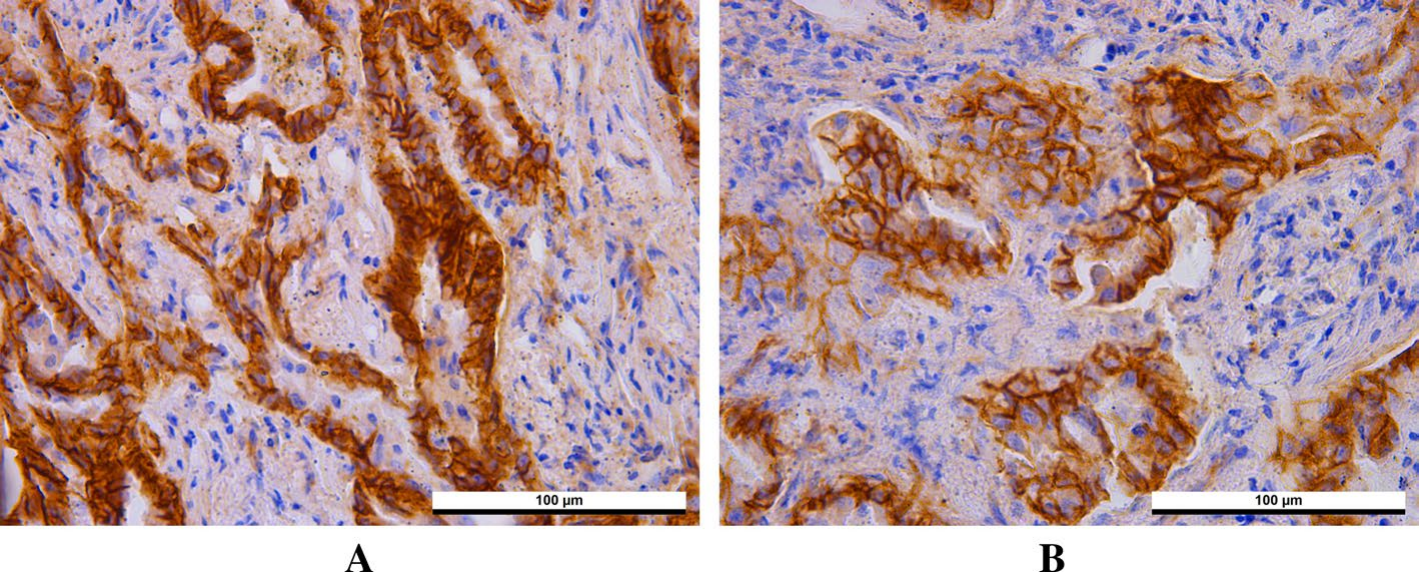
The expression of β-catenin in lung cancer and para-cancerous tissues (Magnification, × 400). β-catenin was abnormally expressed in lung cancer tissues (**A**), but normally expressed in para-cancerous tissues (**B**). The chi-square test was used to assess IHC results

Immunohistochemistry was used to detect the expression of β-catenin in lung tissues. The β-catenin protein was mainly expressed in the cell membrane of the matched para-cancerous tissues. However, β-catenin was abnormally expressed in lung cancer tissues, including reduced expression or loss expression in the cell membrane, cytoplasm or nuclear translocation (Fig. 6). Immunohistochemical results revealed that the high expression rate and abnormal expression rate of β-catenin in cancer tissues was significantly higher than that in para-cancerous tissues (Table 1). We also analyzed the correlation between β-catenin expression and clinicopathological features, and the results indicated that β-catenin expression was not associated with the patient’s sex, age, tumor size or differentiation degree (*P* > 0.05).

**Table 1:** The expression of β-catenin in cancer and para-cancerous tissues

Tissues	N	β-catenin protein expression		
		Positive expression n(%)	High expression n(%)	Abnormal expression n(%)
Para-cancerous tissues	29^a^	17(58.6%)	6(20.7%)	2(6.9%)
Cancer tissues	36^b^	27(75%)	19(52.8%)	25(69.4%)
χ^2^	–	1.970	6.987	25.876
*P*	–	0.160	0.008	0.000

^a^9 detachments of the tissue sections from the slides

^b^2 detachments of the tissue sections from the slides

## Discussion

Invasion and metastasis of NSCLC cancer is a major cause of patient death [22]. At present, EMT is considered to be one of the most common mechanisms in the process of metastasis of cancer cells, and may also be an early event of lung cancer [8]. Consequently, it is helpful for the treatment of NSCLC patients to explore an agent that can effectively inhibit NSCLC cells proliferation, invasion, and metastasis. Studies have shown that fucoxanthin can inhibit the invasion and metastasis of melanoma cells [23], but there are few studies on fucoxanthin in the invasion and metastasis of lung cancer. In the present study, we illustrated that fucoxanthin significantly inhibited the proliferation, metastasis and EMT of LUAD cells.

Apoptosis is a type of mechanisms of cell death, which can be regulated by extrinsic and intrinsic pathway [24, 25]. What is more, the cleavage of PARP and caspase-3, the increase of apoptotic proteins (such as Bax) and the decrease of anti-apoptotic proteins (such as Bcl-2) involve in this apoptosis process [26, 27]. Combined with cell viability results that fucoxanthin inhibited the proliferation of A549 and NCI-H1299 cells, we investigated the effect of fucoxanthin on apoptosis-related proteins of NSCLC cells. The results from the present study indicated that the expression of Bax, Cleaved Caspase 3 and Cleaved PARP increased while Bcl-2, Caspase 3 and PARP decreased after the treatment of fucoxanthin in A549 and NCI-H1299 cells. Thus, we consider that the upregulation of Bax/Bcl-2 ratio and cleavage of PARP and caspase-3 may be an important cause of fucoxanthin in inducing apoptosis and inhibiting cell growth of NSCLC.

TGF-β plays a complex role in tumorigenesis and development. TGF-β can inhibit the growth of tumors and induce cell apoptosis at the early stage of carcinogenesis, whereas it also can promote cancer progression, metastasis, and EMT at later stages of tumor progression [28]. Based on this, we used TGF-β1 to stimulate cells in order to induce EMT in A549 and NCI-H1299 cells. The results demonstrated that TGF-β1 caused remarkable morphologic changes of A549 and NCI-H1299 cells, as well as the decreased expression of E‑cadherin and the increased expression of vimentin. Simultaneously, Wound-healing and Transwell assays showed that TGF-β1 promoted the migration and invasion of NSCLC cells. These findings demonstrated that exposure to TGF-β1 led to EMT, migration and invasion in A549 and NCI-H1299 cells, which is consistent with the previous study [29]. However, fucoxanthin reversed the aforementioned EMT-related protein expression, migration and invasion induced by TGF-β1 in these cells. These results indicate that fucoxanthin may potentially inhibit the metastasis of human NSCLC and may be used as a therapeutic agent for adjuvant therapy. Although fucoxanthin has been shown to inhibit EMT in vitro, the effect of fucoxanthin in vivo may be different. Therefore, future studies will further explore the efficacy of fucoxanthin in reducing distant metastasis in animal models.

Current studies have shown that Wnt/β-catenin signaling pathway may be closely related to EMT, invasion and metastasis of tumors [30, 31]. β-catenin is a critical protein in Wnt/β-catenin signaling pathway. When Wnt ligands bound with Frizzled and Low-density lipoprotein receptors, β-catenin translocates into the nucleus to activate the corresponding transcription factors, and then participates in the process of EMT [32]. In order to investigate the underlying mechanisms of fucoxanthin in EMT of NSCLC cells, we tested the expression of the critical protein β-catenin in the Wnt/β-catenin pathway. We found that TGF-β1 upregulated the expression of the critical protein β-catenin, while the β-catenin expression upregulated by TGF-β1 was reduced by fucoxanthin. We next selected the specific inhibitor XAV939 of Wnt/β-catenin signaling pathway to modulate Wnt signaling. The results showed that XAV939 led to reduced expression of β-catenin and EMT-related proteins which were increased by TGF-β1, and inhibited migration and invasion of A549 and NCI-H1299 cells. Furthermore, we observed that fucoxanthin and XAV939 have similar effect on β-catenin, EMT protein and cell motility. Meanwhile, EMT progression is positively correlated with Wnt/β-catenin pathway activity, indicating that the inhibition of fucoxanthin on EMT progression and cell motility may be associated with reversing Wnt/β-catenin pathway.

As a critical molecule in Wnt/β-catenin signaling pathway, the abnormal activation of β-catenin plays an significant role in EMT and metastasis [33]. In addition, the expression of β-catenin on cell membrane is regarded as a kind of intercellular adhesion molecule, which participates in the regulation of intercellular adhesion and has a negative inhibitory effect on tumor growth [34]. Y. Yang et al.[35] found that the accumulation of β-catenin in nucleus or loss expression in cell membrane would affect the survival of lung cancer patients. Therefore, different localization of β-catenin in cells plays different roles, and its abnormal expression plays a significant role in the occurrence and development of cancer. Our results in vitro suggested that β-catenin was expressed in A549 and NCI-H1299 cells. We further used immunohistochemistry to detect the difference of β-catenin expression in lung cancer tissues and corresponding para-cancerous tissues. The results showed that β-catenin was mainly abnormally expressed in the cytoplasm or nucleus of cancer tissues, while was mainly expressed in the cell membrane of para-cancerous tissues. These data suggested that abnormal expression of β-catenin may play a role in the occurrence and development of lung cancer. Moreover, the high expression rate of β-catenin in lung cancer tissues was higher than that in para-cancerous tissues, suggesting that the detection of β-catenin expression may play a predictive role in the occurrence of lung cancer. On account of this, we will further follow up these patients to observe their prognosis, recurrence and metastasis, and investigate whether the aberrant expression of β-catenin is associated with higher risk of recurrence and metastasis after surgery.

## Conclusion

The results of the present study demonstrated that fucoxanthin inhibited NSCLC cell growth by inducing apoptosis and reversed TGF-β1-induced EMT which might be mediated through inhibiting Wnt/β-catenin pathway. Besides, the analysis of the patients' tissue samples revealed that β-catenin might play a predictive role in the occurrence of lung cancer. Therefore, fucoxanthin may be a potentially effective anti-tumor agent for the treatment of LUAD patients.

## Supplementary Information

Additional file1 (ZIP 2087 KB)

## Data Availability

The datasets used and/or analyzed during the current study are available from the corresponding author on reasonable request. All data generated or analyzed during this study are included in this published article.
