# Supplementary figures and images for: Fucoxanthin induces apoptosis and reverses epithelial-mesenchymal transition via inhibiting Wnt/β-catenin pathway in lung adenocarcinoma
Source: Discov Oncol. 2022 Oct 3;13:98. doi: 10.1007/s12672-022-00564-4 (PMC9530106; doi:10.1007/s12672-022-00564-4)

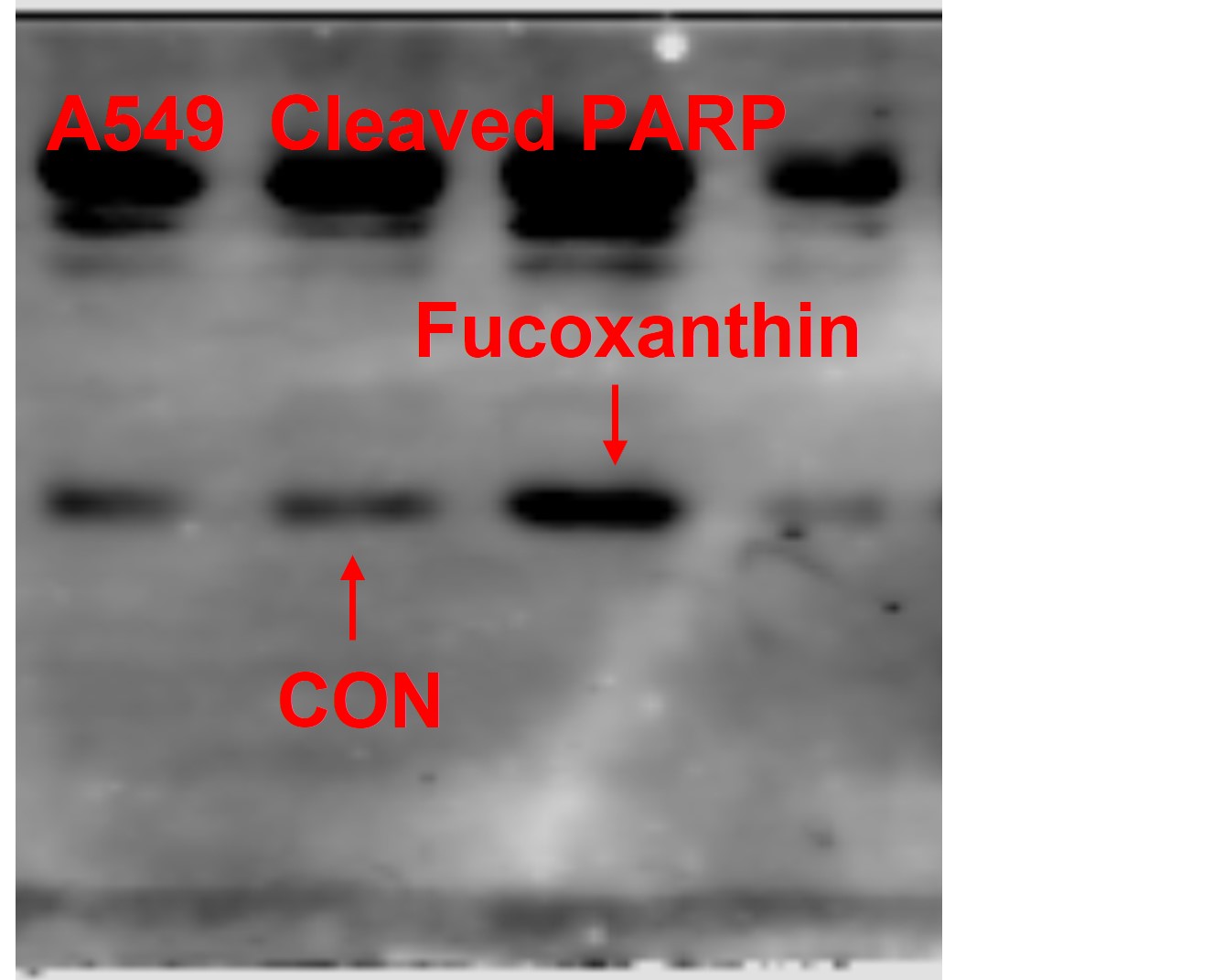

Supplement: Supplementary file 1 — Additional file1 (ZIP 2087 KB) [file 12672_2022_564_MOESM1_ESM.zip › WB/fig 2a(A549 Cleaved PARP).jpg]

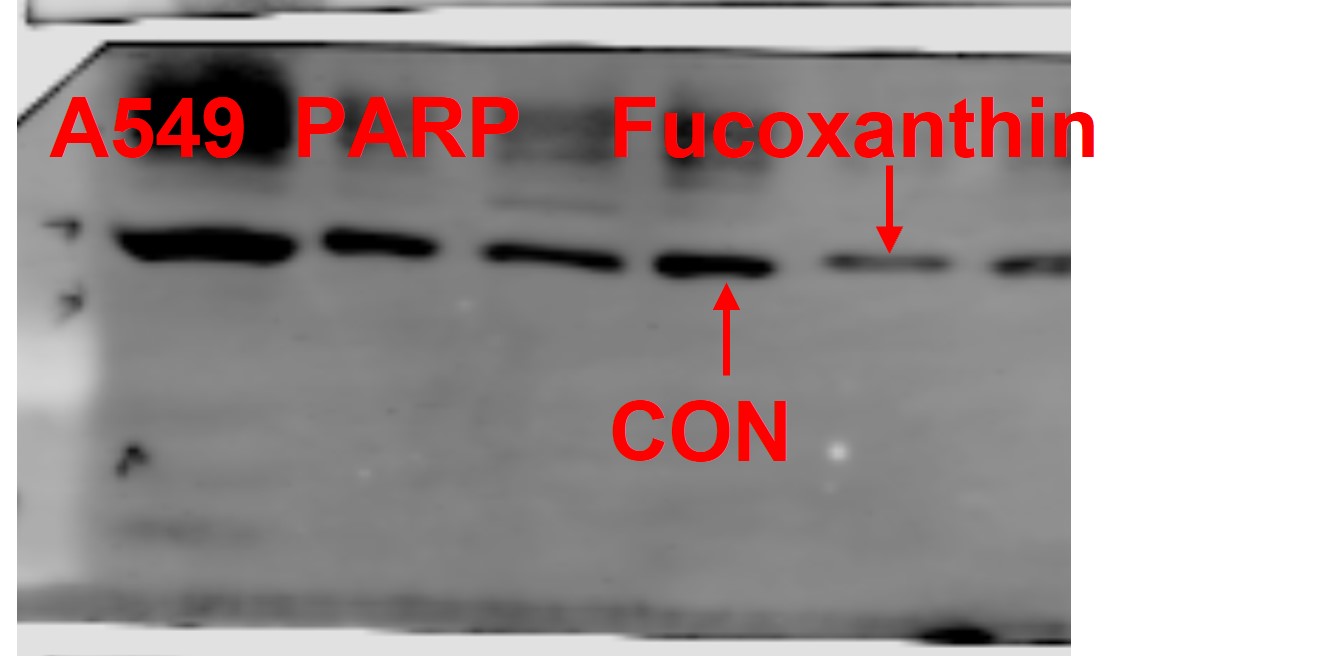

Supplement: Supplementary file 1 — Additional file1 (ZIP 2087 KB) [file 12672_2022_564_MOESM1_ESM.zip › WB/fig 2a(A549 PARP).jpg]

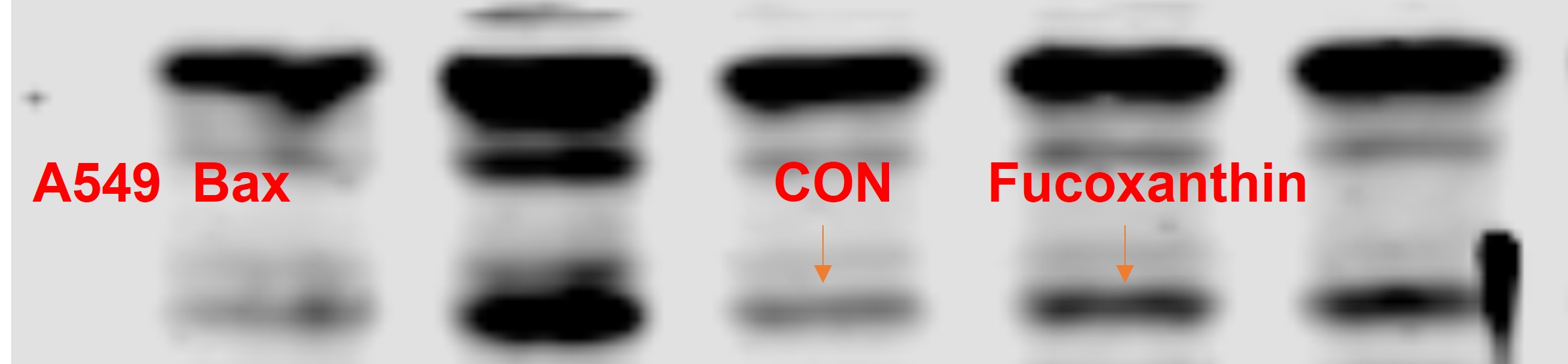

Supplement: Supplementary file 1 — Additional file1 (ZIP 2087 KB) [file 12672_2022_564_MOESM1_ESM.zip › WB/fig 2a(A549 Bax).jpg]

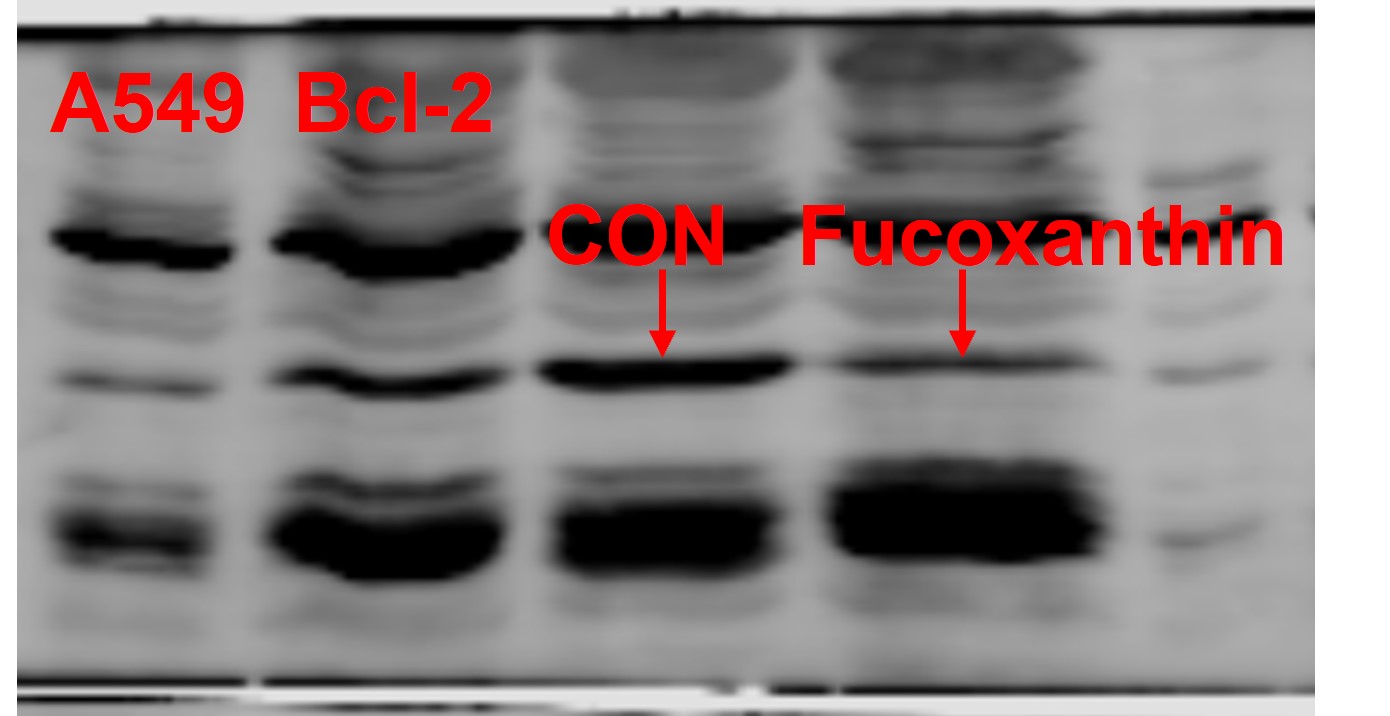

Supplement: Supplementary file 1 — Additional file1 (ZIP 2087 KB) [file 12672_2022_564_MOESM1_ESM.zip › WB/fig 2a(A549 Bcl-2).jpg]

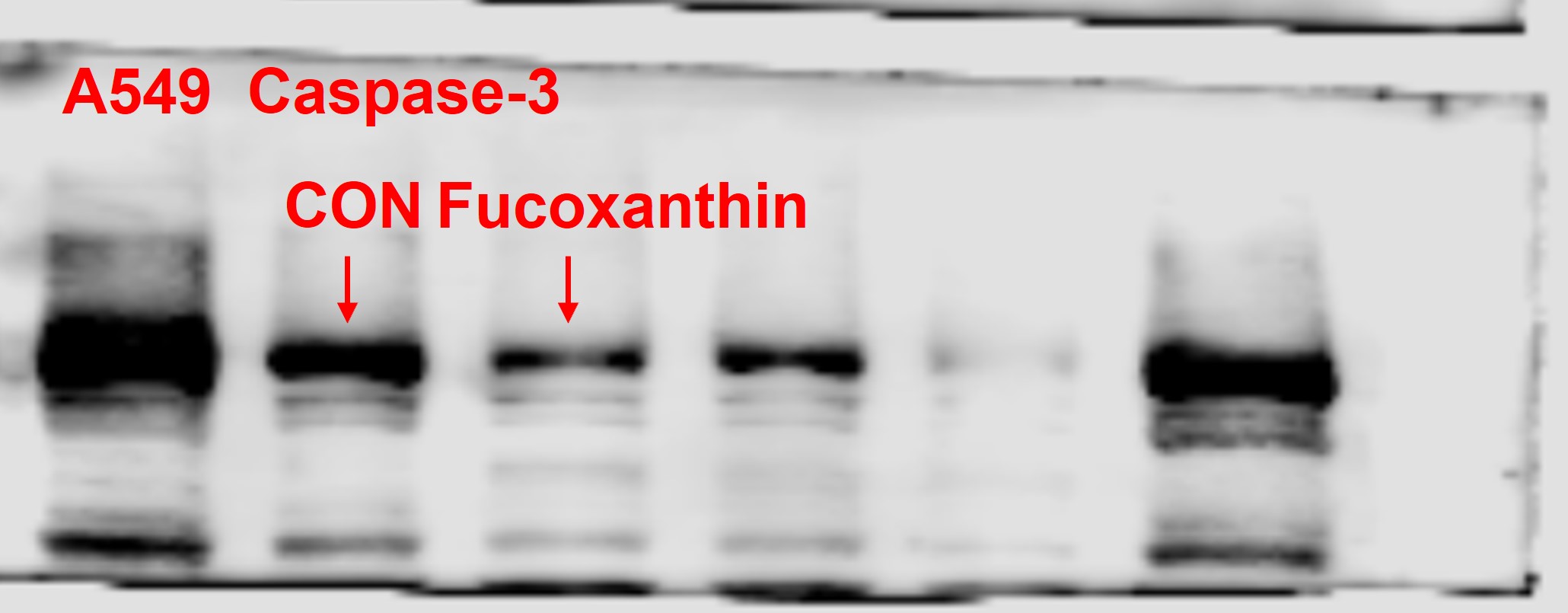

Supplement: Supplementary file 1 — Additional file1 (ZIP 2087 KB) [file 12672_2022_564_MOESM1_ESM.zip › WB/fig 2a(A549 Caspase-3).jpg]

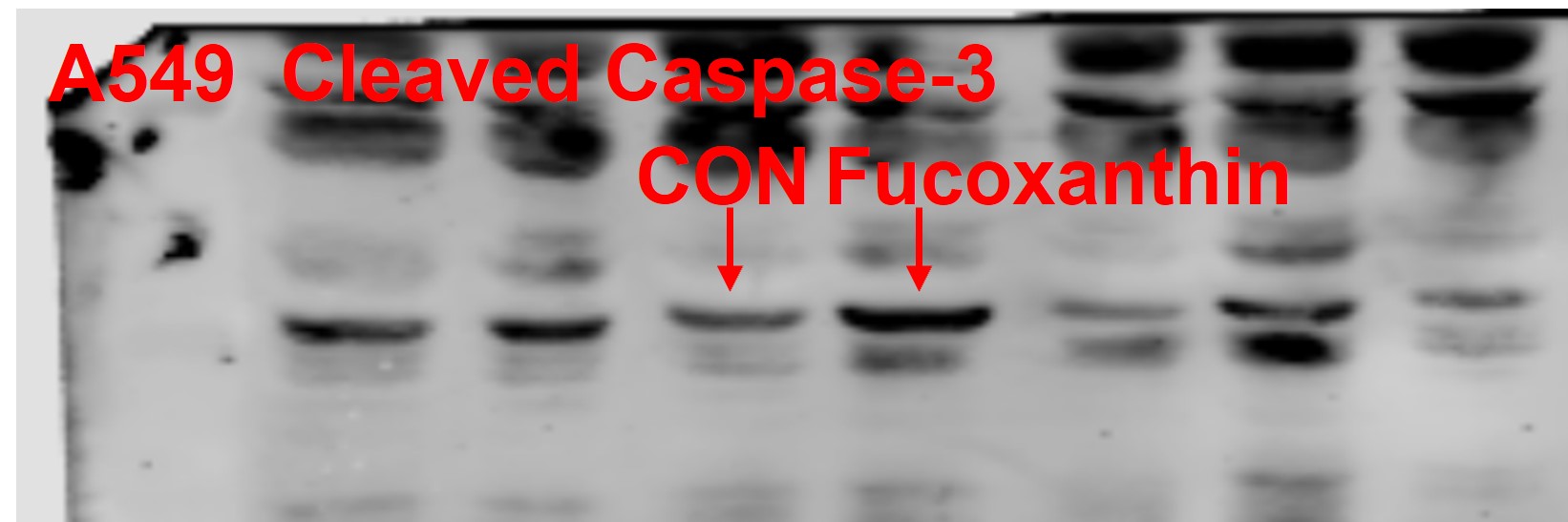

Supplement: Supplementary file 1 — Additional file1 (ZIP 2087 KB) [file 12672_2022_564_MOESM1_ESM.zip › WB/fig 2a(A549 Cleaved Caspase-3).jpg]

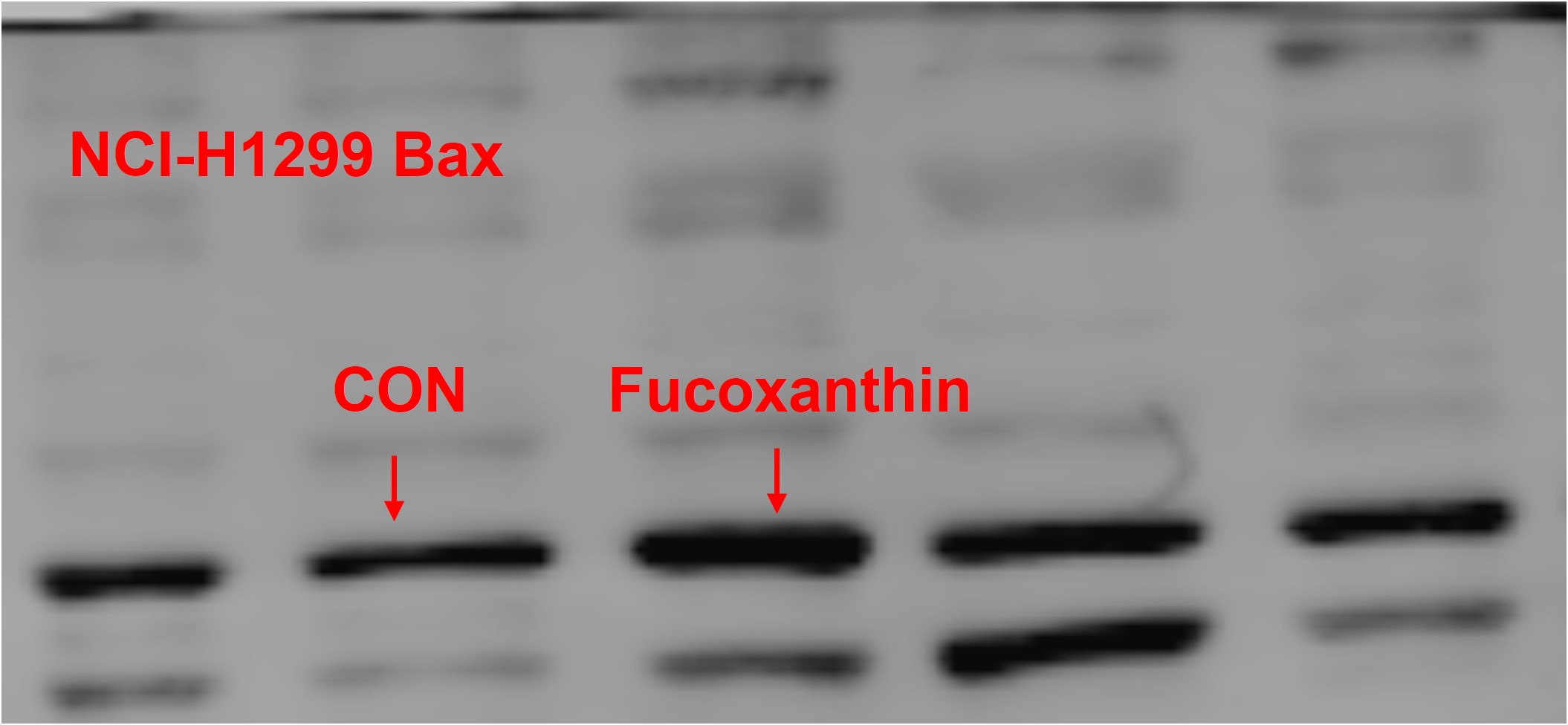

Supplement: Supplementary file 1 — Additional file1 (ZIP 2087 KB) [file 12672_2022_564_MOESM1_ESM.zip › WB/fig 2a(NCI-H1299 Bax).jpg]

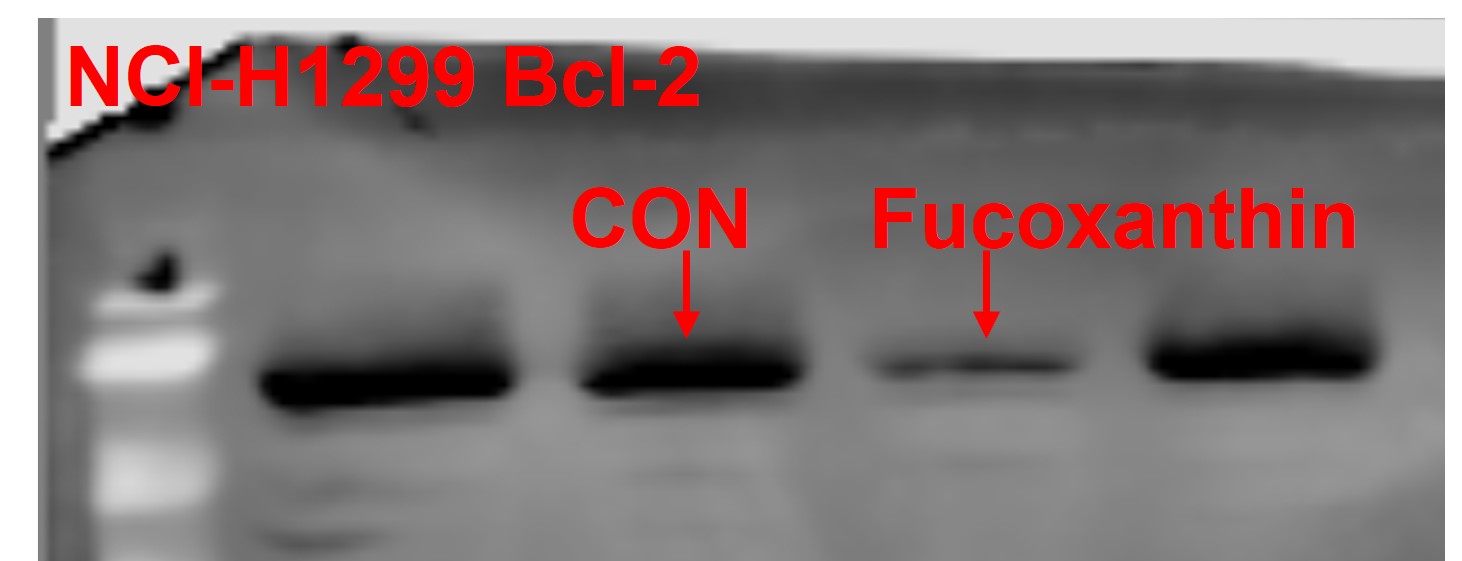

Supplement: Supplementary file 1 — Additional file1 (ZIP 2087 KB) [file 12672_2022_564_MOESM1_ESM.zip › WB/fig 2a(NCI-H1299 Bcl-2).jpg]

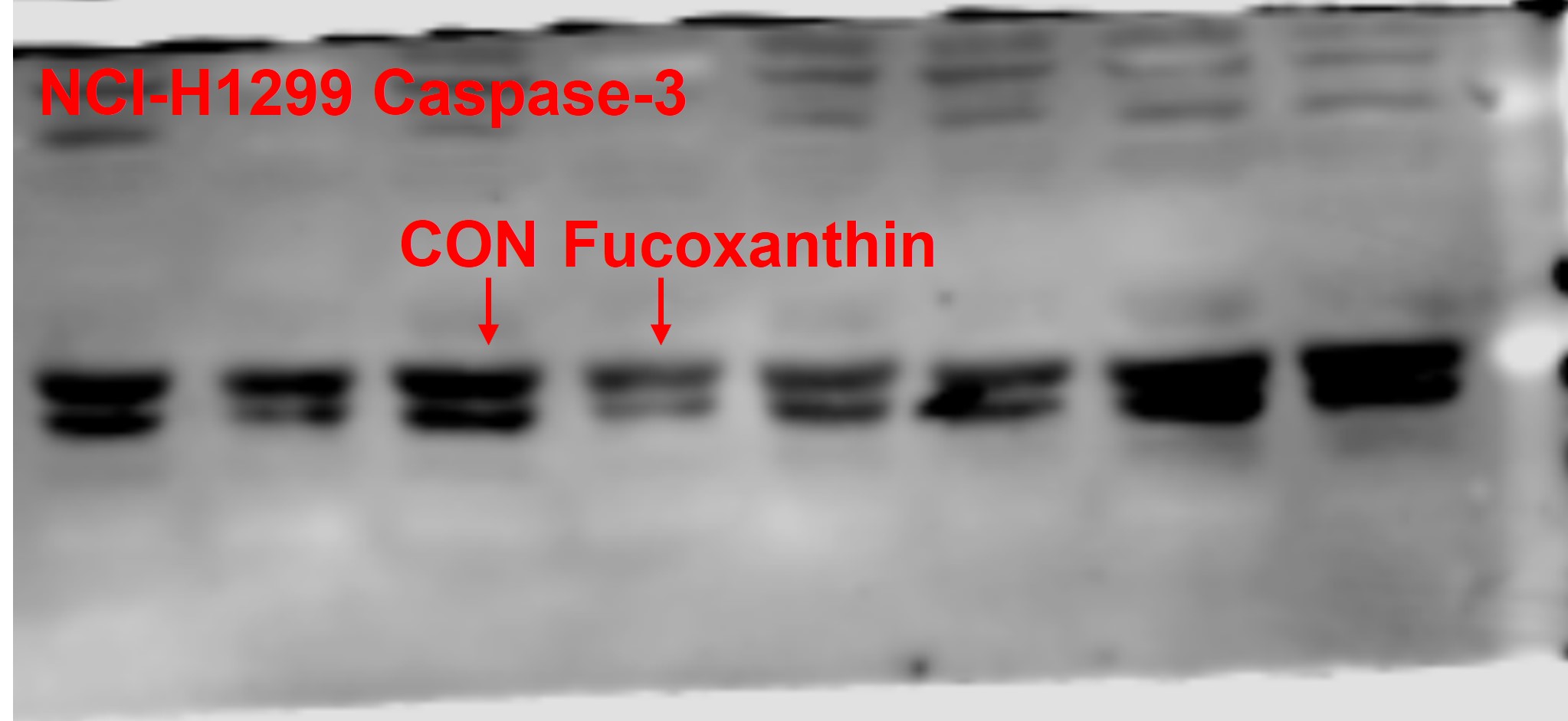

Supplement: Supplementary file 1 — Additional file1 (ZIP 2087 KB) [file 12672_2022_564_MOESM1_ESM.zip › WB/fig 2a(NCI-H1299 Caspase-3).jpg]

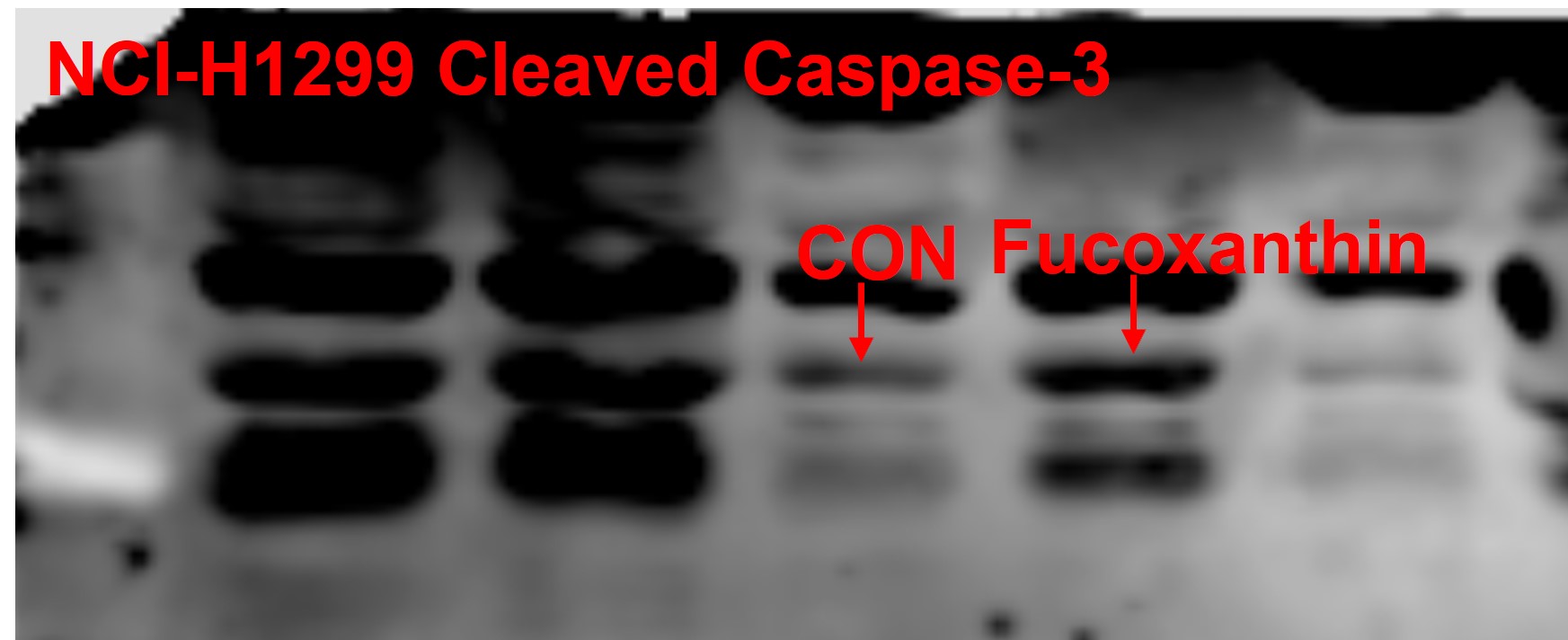

Supplement: Supplementary file 1 — Additional file1 (ZIP 2087 KB) [file 12672_2022_564_MOESM1_ESM.zip › WB/fig 2a(NCI-H1299 Cleaved Caspase-3).jpg]

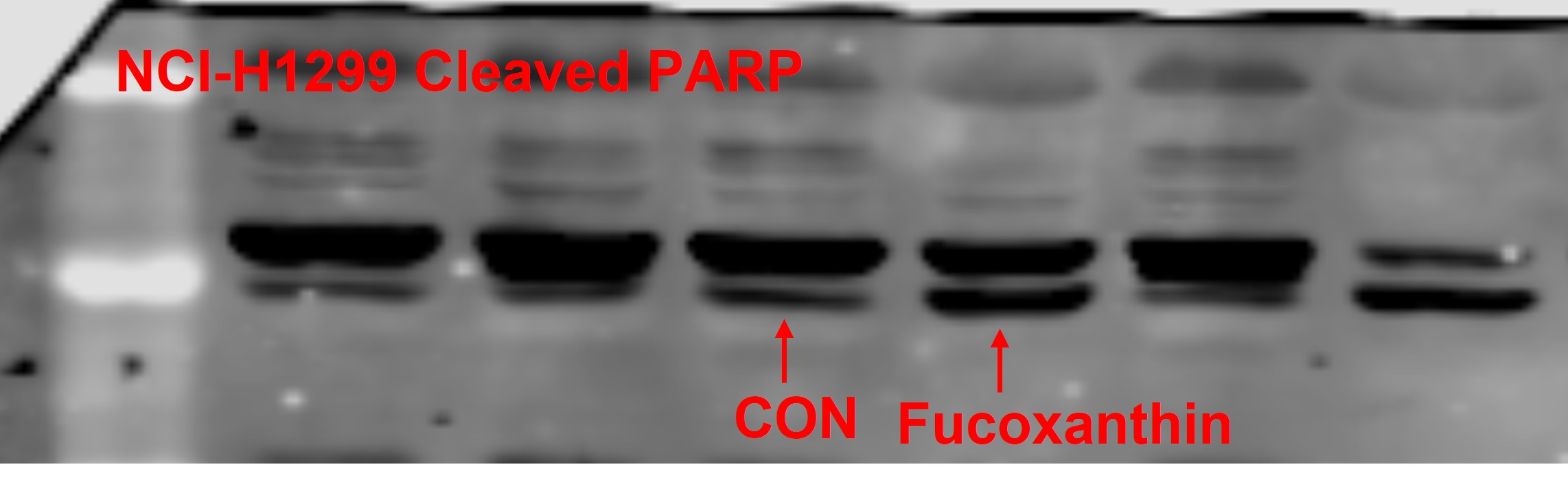

Supplement: Supplementary file 1 — Additional file1 (ZIP 2087 KB) [file 12672_2022_564_MOESM1_ESM.zip › WB/fig 2a(NCI-H1299 Cleaved PARP).jpg]

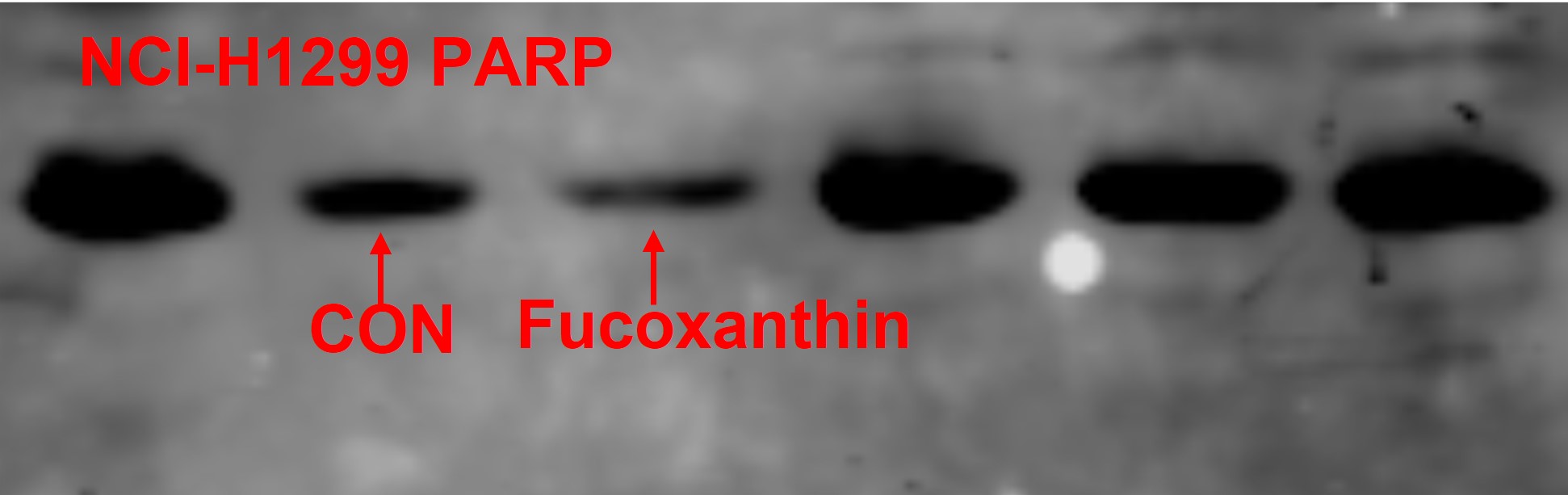

Supplement: Supplementary file 1 — Additional file1 (ZIP 2087 KB) [file 12672_2022_564_MOESM1_ESM.zip › WB/fig 2a(NCI-H1299 PARP).jpg]

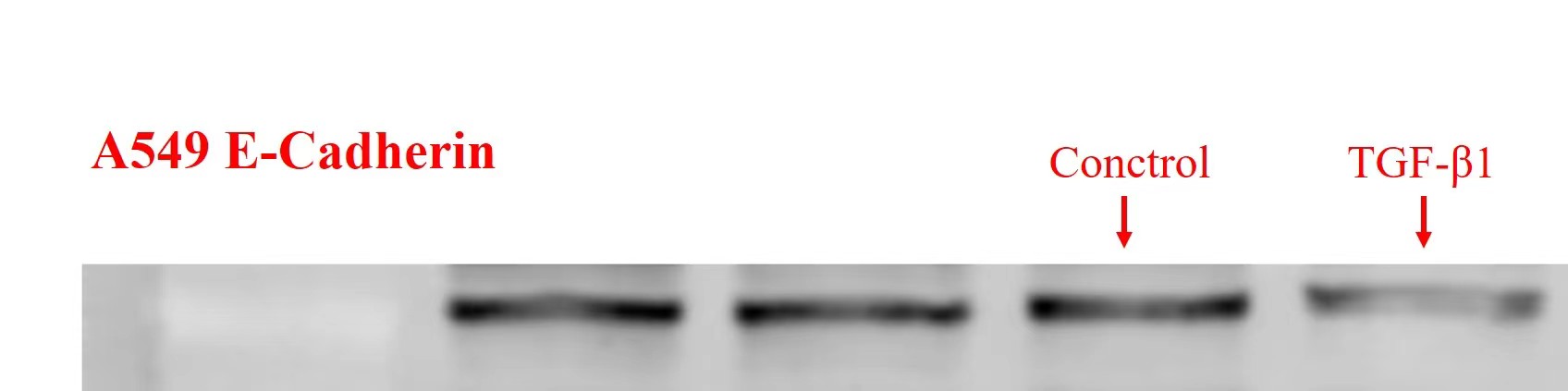

Supplement: Supplementary file 1 — Additional file1 (ZIP 2087 KB) [file 12672_2022_564_MOESM1_ESM.zip › WB/fig 3b(A549 E-Cadherin).jpg]

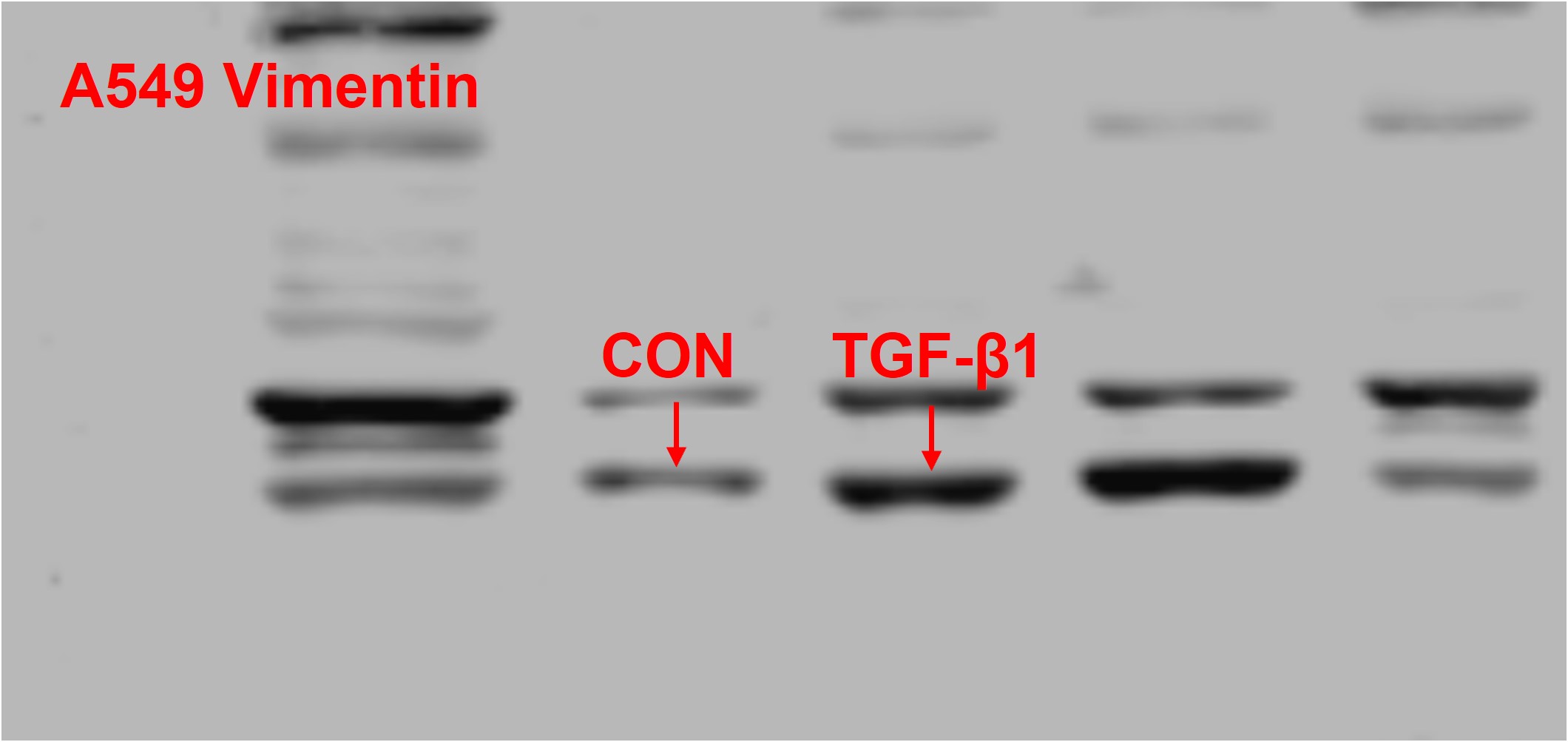

Supplement: Supplementary file 1 — Additional file1 (ZIP 2087 KB) [file 12672_2022_564_MOESM1_ESM.zip › WB/fig 3b(A549 Vimentin).jpg]

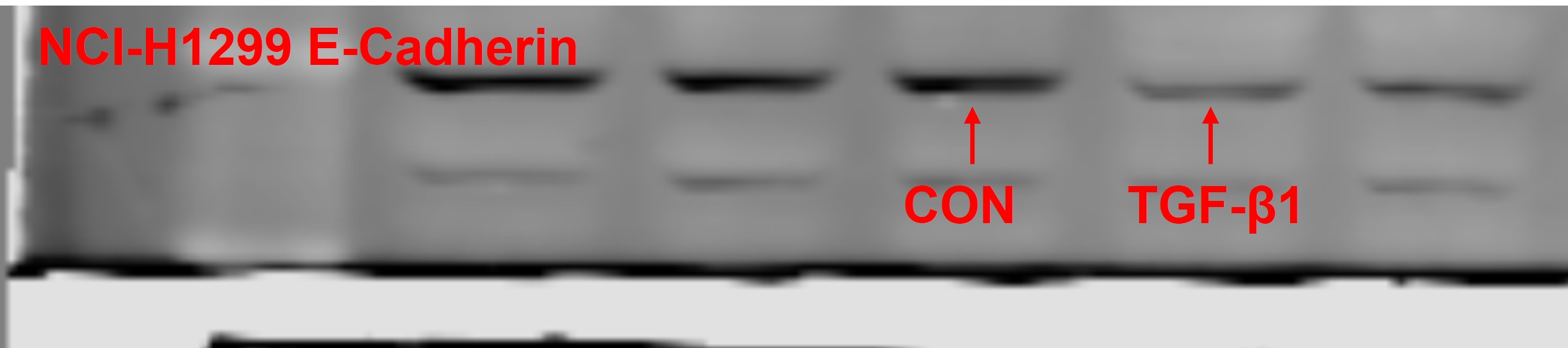

Supplement: Supplementary file 1 — Additional file1 (ZIP 2087 KB) [file 12672_2022_564_MOESM1_ESM.zip › WB/fig 3b(NCI-H1299 E-Cadherin).jpg]

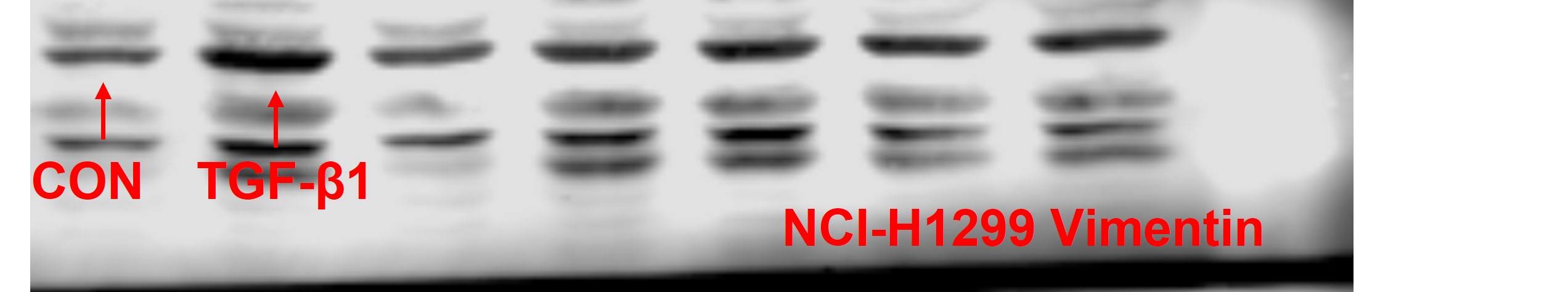

Supplement: Supplementary file 1 — Additional file1 (ZIP 2087 KB) [file 12672_2022_564_MOESM1_ESM.zip › WB/fig 3b(NCI-H1299 Vimentin).jpg]

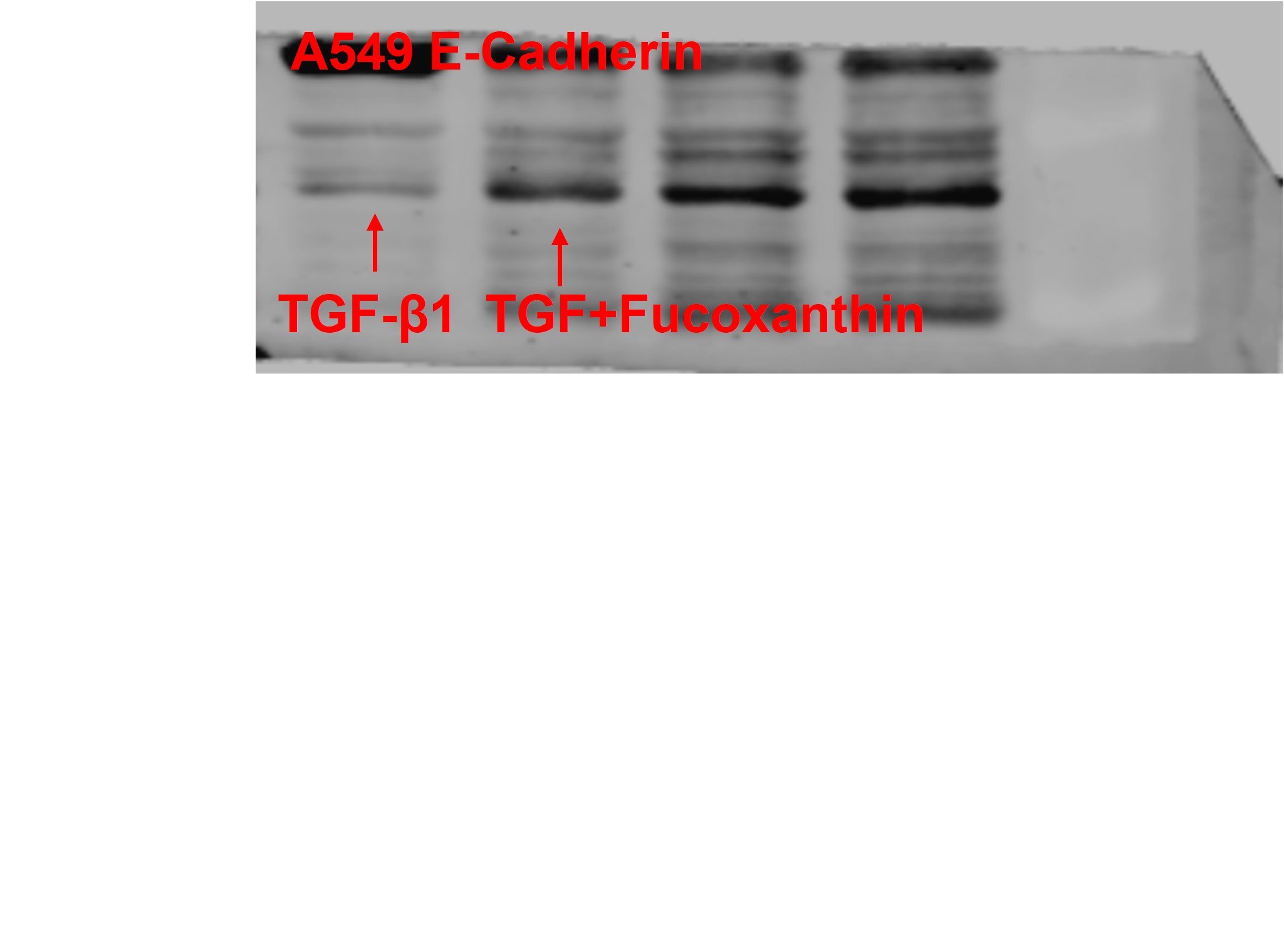

Supplement: Supplementary file 1 — Additional file1 (ZIP 2087 KB) [file 12672_2022_564_MOESM1_ESM.zip › WB/fig 4a(A549 E-Cadherin).jpg]

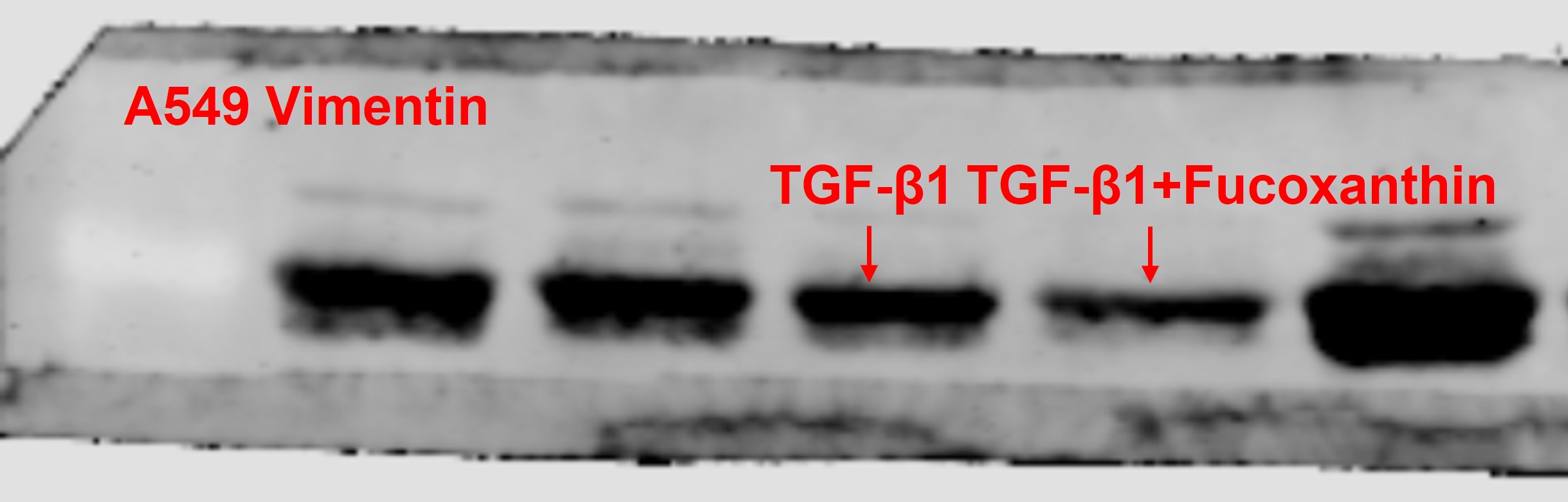

Supplement: Supplementary file 1 — Additional file1 (ZIP 2087 KB) [file 12672_2022_564_MOESM1_ESM.zip › WB/fig 4a(A549 Vimentin).jpg]

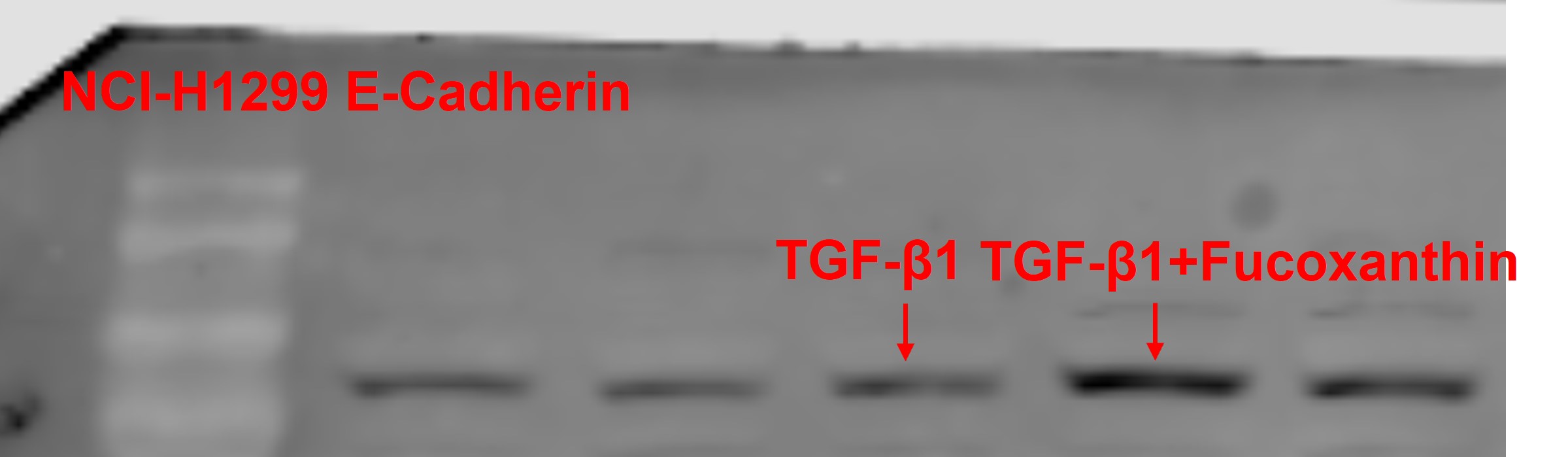

Supplement: Supplementary file 1 — Additional file1 (ZIP 2087 KB) [file 12672_2022_564_MOESM1_ESM.zip › WB/fig 4a(NCI-H1299 E-Cadherin).jpg]

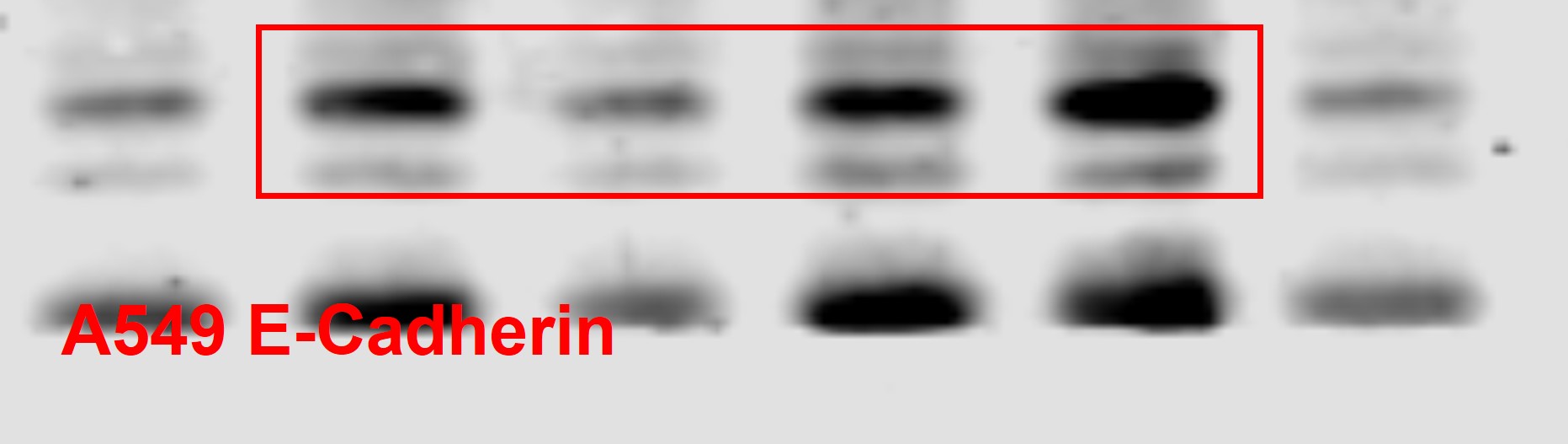

Supplement: Supplementary file 1 — Additional file1 (ZIP 2087 KB) [file 12672_2022_564_MOESM1_ESM.zip › WB/fig 5a(A549 E-Cadherin).jpg]

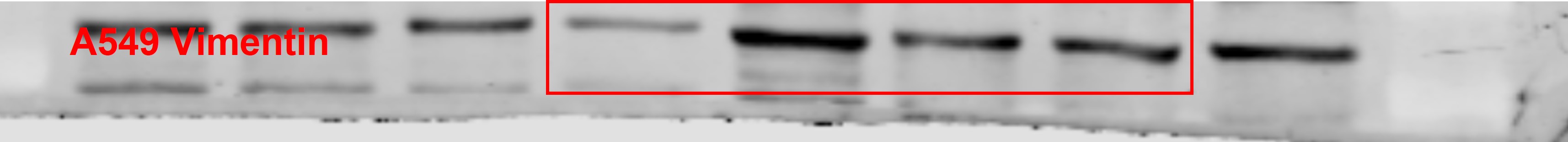

Supplement: Supplementary file 1 — Additional file1 (ZIP 2087 KB) [file 12672_2022_564_MOESM1_ESM.zip › WB/fig 5a(A549 Vimentin).jpg]

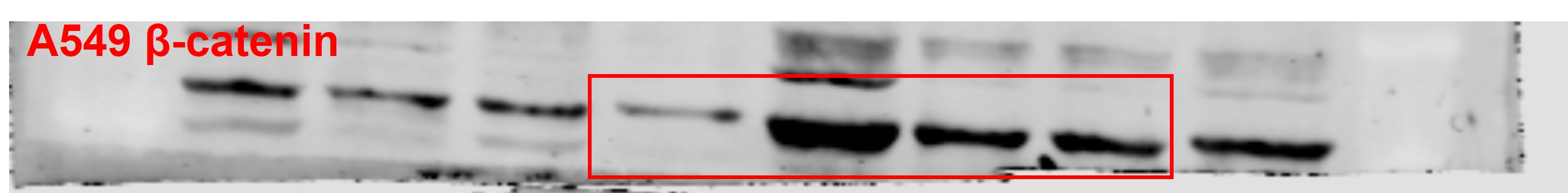

Supplement: Supplementary file 1 — Additional file1 (ZIP 2087 KB) [file 12672_2022_564_MOESM1_ESM.zip › WB/fig 5a(A549 β-catenin).jpg]

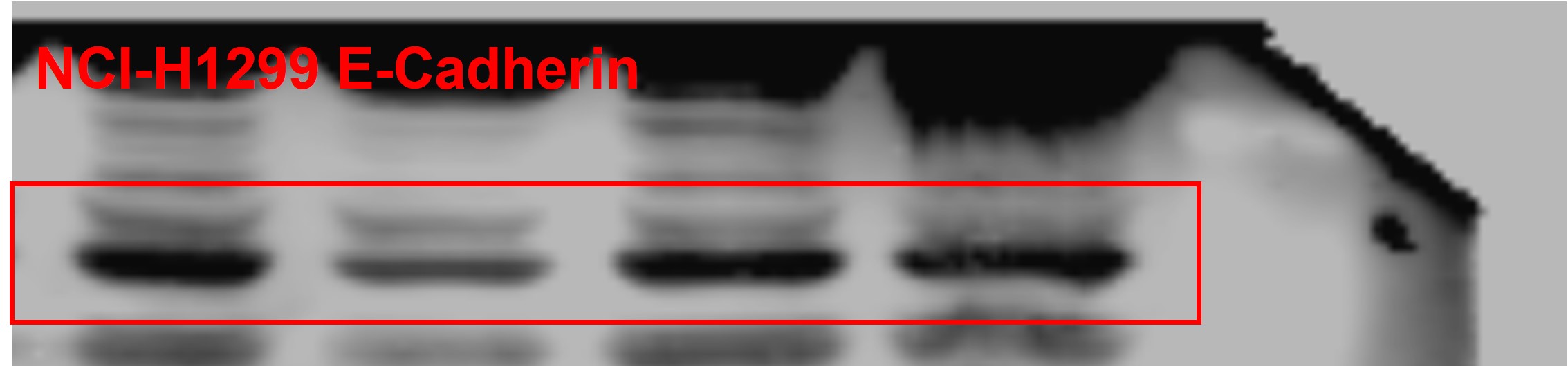

Supplement: Supplementary file 1 — Additional file1 (ZIP 2087 KB) [file 12672_2022_564_MOESM1_ESM.zip › WB/fig 5a(NCI-H1299 E-Cadherin).jpg]

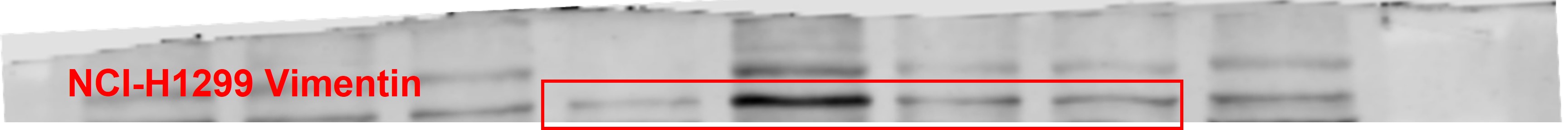

Supplement: Supplementary file 1 — Additional file1 (ZIP 2087 KB) [file 12672_2022_564_MOESM1_ESM.zip › WB/fig 5a(NCI-H1299 Vimentin).jpg]
